# In aged mice, low surrogate light chain promotes pro-B-cell apoptotic resistance, compromises the PreBCR checkpoint, and favors generation of autoreactive, phosphorylcholine-specific B cells

**DOI:** 10.1111/acel.12302

**Published:** 2015-02-27

**Authors:** Michelle Ratliff, Sarah Alter, Kelly McAvoy, Daniela Frasca, Jacqueline A Wright, Sandra S Zinkel, Wasif N Khan, Bonnie B Blomberg, Richard L Riley

**Affiliations:** 1 Department of Microbiology & Immunology, University of Miami Miller School of MedicineMiami, FL, 33136, USA; 2 Department of Medicine, Division of Hematology/Oncology, Vanderbilt University School of MedicineNashville, TN, 37232, USA

**Keywords:** aging, autoreactivity, B cells, B lymphopoeisis, inflammation, phosphorylcholine, senescence, TNF alpha

## Abstract

In aged mice, new B-cell development is diminished and the antibody repertoire becomes more autoreactive. Our studies suggest that (i) apoptosis contributes to reduced B lymphopoiesis in old age and preferentially eliminates those B-cell precursors with higher levels of the surrogate light chain (SLC) proteins (λ5/VpreB) and (ii) λ5^low^ B-cell precursors generate new B cells which show increased reactivity to the self-antigen/bacterial antigen phosphorylcholine (PC). Pro-B cells in old bone marrow as well as pro-B cells from young adult λ5-deficient mice are resistant to cytokine-induced apoptosis (TNFα; TGFβ), indicating that low λ5 expression in pro-B cells is sufficient to cause increased survival. Transfer of TNFα-producing ‘age-associated B cells’ (ABC; CD21/35^−^ CD23^−^) or follicular (FO) B cells from aged mice into RAG-2 KO recipients led to preferential loss of λ5^high^ pro-B cells, but retention of λ5^low^, apoptosis-resistant pro-B cells. In old mice, there is increased reactivity to PC in both immature bone marrow B cells and mature splenic FO B cells. In young mice, absence of λ5 expression led to a similar increase in PC reactivity among bone marrow and splenic B cells. We propose that in old age, increased apoptosis, mediated in part by TNFα-producing B cells, results in preferential loss of SLC^high^ pro-B cells within the bone marrow. Further B-cell development then occurs via an ‘SLC^low^’ pathway that not only impairs B-cell generation, but promotes autoreactivity within the naïve antibody repertoires in the bone marrow and periphery.

## Introduction

In murine senescence, B lymphopoiesis is highly compromised with losses at multiple developmental stages (Stephan *et al*., 1996; Van der Put *et al*., 2003; Cancro *et al*., 2009). In old mice representing a variety of inbred strains, both pre-B cells and pro-B cells are reduced although there is significant individual variation in the extent of B-cell precursor loss (Van der Put *et al*., 2003). While some loss in pre-B cells is typical in old mice, the incidence of individuals which show both extensive loss of pre-B cells (>70%) and partial loss of pro-B cells (∼50%) increases with chronological old age (Van der Put *et al*., 2003). In old mice, particularly those with partial loss of pro-B cells, the residual pro-B cells have diminished levels of the surrogate light chain (SLC) proteins λ5 and VpreB (Sherwood *et al*., 1998, 2000; Alter-Wolf *et al*., 2009). This coincides with decreases in expression of E47/E2A (Sherwood *et al*., 2000; Frasca *et al*., 2003; Van der Put *et al*., 2004; King *et al*., 2007) as well as Early B-cell Factor (EBF) (Lescale *et al*., 2010; Riley, 2013). Both E2A and EBF regulate expression of the surrogate light chain genes (Sigvardsson *et al*., 1997).

While reduced E2A/EBF provides an explanation for low SLC in old B-cell precursors, the cellular mechanisms within the bone marrow microenvironment which promote loss of significant numbers of B-cell precursors in old age and induce the ‘low SLC’ phenotype remain unclear. The reduced expression of surrogate light chain in B-cell precursors likely disrupts normal pre-B-cell receptor (preBCR) expression and function in old mice. This in turn would be expected to affect the ‘readout’ of the antibody repertoire, as SLC affects V_H_ expression and selection in pre-B and B cells (Ye *et al*., 1996; ten Boekel *et al*., 1997; Kline *et al*., 1998; Kawano *et al*., 2006). Moreover, in the absence of SLC, autoantibody frequencies are increased, suggesting that, in part, central tolerance is affected by SLC levels (Keenan *et al*., 2008). While alterations in the antibody repertoires of aged mice in both immature and mature B-cell compartments have been described (Zharhary & Klinman, 1986; Riley *et al*., 1989), whether low SLC expression in aged B-cell precursors contributes to altered antibody repertoire ‘readout’ has not been assessed.

In this report, we demonstrate that levels of the SLC protein λ5 determine the relative susceptibility of pro-B cells to apoptosis induced by two cytokines, TNFα and TGFβ, which act via different signaling pathways (Kee *et al*., 2001; Gupta & Gollapudi, 2005; Ramesh *et al*., 2009). Reduced expression of λ5 protein also affects the readout of the immature B-cell repertoire as evidenced by changes in the reactivity of newly generated bone marrow B cells in aged mice, as well as young λ5 gene KO mice, to the self (and bacterial) epitope phosphorylcholine (PC).

Increased apoptosis among B-cell precursors in aged mice has been reported (Kirman *et al*., 1998; Van der Put *et al*., 2003). However, the mechanisms which heighten apoptosis in the bone marrow of old mice remain to be determined. Recently, it has been proposed that peripheral B cells in aged mice provide negative ‘feedback signals which reduce B lymphopoiesis in the bone marrow (Keren *et al*., 2011a,b). We and others have shown that a novel mature B-cell subset, the ‘age-associated B cells (ABC)’, accumulates in the spleens as well as the bone marrow of old mice (Hao *et al*., 2011; Ratliff *et al*., 2013) while follicular (FO) B cells decline.

Age-associated B cells, as well as FO B cells, secrete the pro-inflammatory cytokine TNFα (Frasca *et al*., 2012; Ratliff *et al*., 2013). Herein, we employ a model of ‘B-cell feedback’, for example, adoptive transfer of aged B cells into RAG-2 KO recipients, to show that aged B cells not only can promote loss of pro-B cells within the bone marrow *in vivo*, but the preferential loss of those bone marrow pro-B cells which are relatively high in λ5 expression. In old age, increased apoptosis may contribute to an overall reduction in B-cell precursors, but maintenance of a subset of pro-B cells with relatively lower SLC. This, consequently, results in alterations in the ‘readout’ of the antibody repertoire in old mice.

## Results

### Residual pro-B cells in aged mice are low in λ5 surrogate light chain and resistant to apoptosis

**Fig 1:**
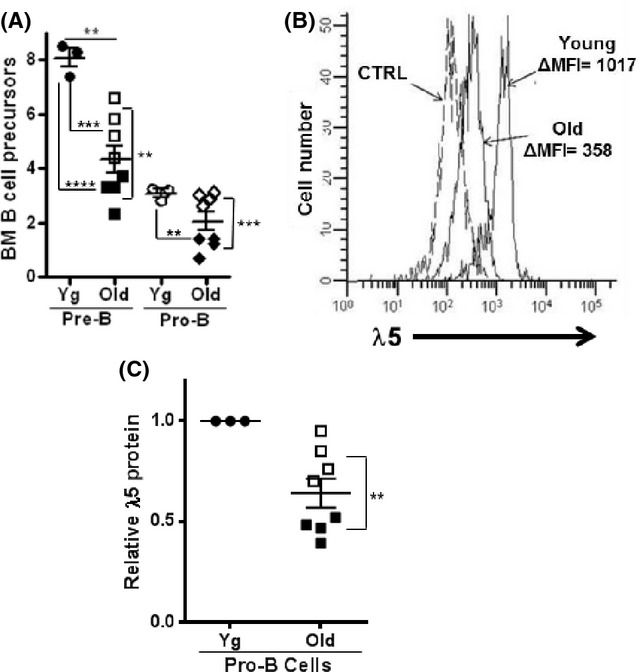
Pro-B cells from aged mice are reduced and have decreased λ5 surrogate light chain (SLC) protein. (A) Bone marrow cells from 3 young (3 months) and 8 aged (22–24 months) BALB/c mice were stained for pro-B cells and pre-B cells and proportions (%) in bone marrow are shown. Two subsets of aged mice were apparent (Van der Put *et al*., 2003): those with low pre-B cells, but normal pro-B cells (‘moderate depletion’ phenotype, white symbols) and those with even lower pre-B cells and partial (∼50%) losses in pro-B cells (‘severe depletion’ phenotype, dark symbols). (B) Cytoplasmic λ5 levels were determined by fluorescence staining with LM34 mAb in pro-B cells in young and old BALB/c mice. A representative histogram typical of aged mice with ‘severe pre-B depletion’ is shown CTRL, isotype control staining. (C) Bone marrow cells from each of the young and aged mice from (A) were stained for cytoplasmic λ5 SLC and Δmean fluorescence intensity values for old pro-B cells are expressed relative to that of young adult controls. Statistical significance (two-tailed Student's *t*-test): **P < 0.004; ***P = 0.0001.

In aged mice, heterogeneity is seen in pro-B and pre-B-cell loss within the aged mouse population and this is seen for both old BALB/c and C57BL/6 mice. For example, some aged mice have low pre-B cells (∼10–50% reductions), but no changes in pro-B-cell numbers (e.g. a ‘moderate depletion phenotype’), while others have even lower pre-B cells and also ∼50% lower pro-B-cell numbers (e.g., a ‘severe depletion phenotype’ (Van der Put *et al*., 2003). This heterogeneity of phenotypes in old mice was replicated in the eight individual old BALB/c mice evaluated in Fig.1A.

As previously indicated (Sherwood *et al*., 1998, 2000; Alter-Wolf *et al*., 2009), and shown in Fig.1B, the remaining pro-B cells in the bone marrow of aged BALB/c mice are low in λ5 SLC and this is also observed for aged B6 mice (data not shown). We found that low λ5 protein expression was seen in pro-B cells from ‘moderately depleted’ aged mice (∼60–80% of young pro-B λ5 levels) and was further reduced in ‘severely depleted’ aged BALB/c mice to ∼30–50% of young pro-B-cell λ5 protein levels (Fig.1C).

**Fig 2:**
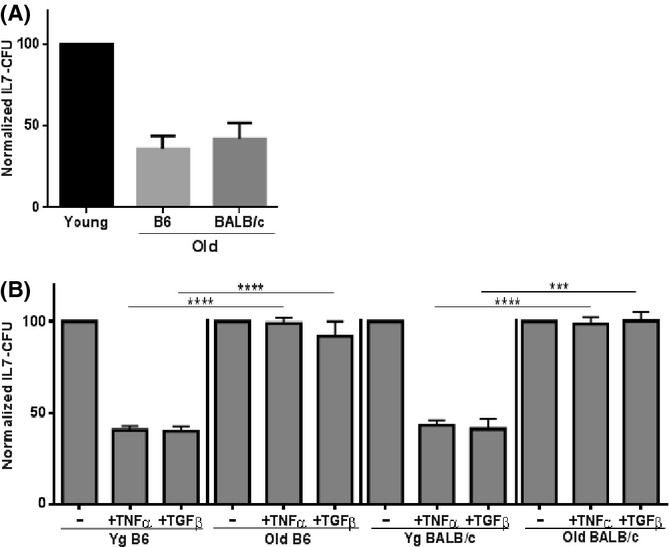
B-cell precursors in aged bone marrow are resistant to cytokine-induced apoptosis. (A) Unfractionated bone marrow cells (1 × 10^6^ ml^−1^) from young and aged ‘severely pre-B depleted’ (see Fig.1) B6 and BALB/c mice were cultured and IL-7 CFU determined. IL-7 CFU from young controls were set at 100%. (B) Bone marrow cells from young and aged mice were cultured with either 10 ng ml^−1^ rmTNFα or 10 ng ml^−1^ rmTGFβ in IL-7 CFU assays. IL-7 CFU for cultures not treated with TNFα or TGFβ were set to 100% for each group. Normalized IL-7 CFU values were obtained for 6–12 individual experiments for each group. Statistical significance (two-tailed Student's *t*-test): ***P = 0.0001; ****P < 0.0001.

As expected, given their reduced pro-B/pre-B cells, IL-7-dependent B-cell precursor growth, as measured by bone marrow IL-7 CFU, is likewise reduced in ‘severely depleted’ aged BALB/c and B6 mice (Fig.2A). IL-7 responsive early pre-B cells are highly resistant to apoptosis induced by cytokines, while pro-B cells are highly susceptible (e.g., ∼80% of pro-B IL-7 CFU is inhibited by TNFα) (Ratliff *et al*., 2013). As pre-B and pro-B cells each contribute about one-half to the total IL-7 CFU response (Ratliff *et al*., 2013), IL-7 CFU from young adult bone marrow were inhibited by ∼60% as expected upon challenge with either TNFα or TGFβ (Fig.2B). Strikingly, however, IL-7 CFU activity in the limited pool of B-cell precursors in old BALB/c and B6 mice was resistant to two apoptosis-inducing cytokines, TNFα and TGFβ (Fig.2B), when used at optimal concentrations (10 ng ml^−1^) for IL-7 CFU inhibition.

**Fig 3:**
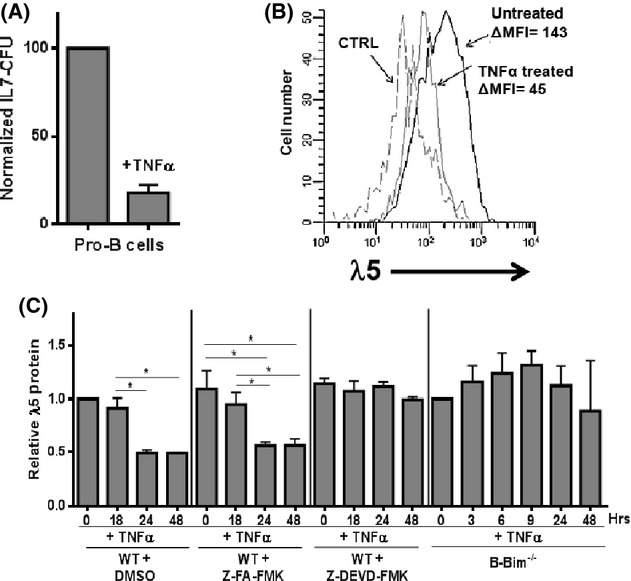
TNFα induces apoptosis preferentially in pro-B cells which express relatively high levels of λ5 surrogate light chain. (A) Bone marrow pro-B cells from young adult (3 months) B6 mice were cultured in IL-7 CFU assays (1 × 10^5^ ml^−1^) with or without 10 ng ml^−1^ rmTNFα. The IL-7-dependent colonies were harvested at day 8. (B) Remaining IL-7 CFU shown in (A) experiments were stained for pro-B-cell markers as well as cytoplasmic λ5. Pro-B-cell cytoplasmic λ5 expression is shown for untreated and rmTNFα-treated groups. CTRL, isotype control staining. Data are representative of five experiments. (C) CD19^+^ cells from young wild-type (WT) or B-Bim knockout mice were cultured with 5 ng ml^−1^ rmIL-7 and DMSO (0.5% final concentration) either alone or with 10 ng ml^−1^ rmTNFα and, where shown, 50 mm Z-FA-FMK (control) in DMSO or 50 mm Z-DEVD-FMK (caspase 3 inhibitor) in DMSO for the times indicated. Harvested B-lineage cells were stained for cytoplasmic λ5 protein with the Δmean fluorescence intensity (MFI) determined. Time zero values for λ5 were set at 1.0. Relative λ5 protein values, based on ΔMFI values, were obtained in 3–4 separate experiments for each group. P values (two-tailed Student's *t*-test) were: *P < 0.05.

Of interest, when young-adult-isolated pro-B cells were treated *in vitro* with TNFα, as expected (Ratliff *et al*., 2013) ∼20% of control IL-7 CFU were observed (Fig.3A); however, the pro-B cells in these remaining colonies had λ5 protein expression which was approximately one-third of that seen normally (Fig.3B). The results of these experiments indicated that pro-B cells which resist TNFα-mediated apoptosis were also low in λ5 SLC. We reasoned that either apoptosis occurs preferentially in those pro-B cells with relatively high λ5 expression, leaving pro-B cells with low λ5 protein relatively unaffected, or that these apoptotic cytokines downregulated λ5 expression in all pro-B cells separate from induction of apoptosis. To distinguish between these possibilities, we blocked apoptosis in young pro-B cells and then assessed λ5 expression in pro-B cells exposed to TNFα. Two methods were used to block apoptosis in pro-B cells: (i) use of caspase inhibitors, as previously described (Ratliff *et al*., 2013) and (ii) use of mice deficient in pro-apoptotic Bim specifically within CD19^+^ B-lineage cells (B-Bim mice).

When B-lineage cells were treated with TNFα over a 48 h period, pro-B-cell numbers were decreased ∼ twofold (data not shown), consistent with prior results (King *et al*., 2009), and λ5 cytoplasmic levels declined by ∼50% (Fig.3C). However, when apoptosis was blocked by either caspase inhibitors (Z-DEVD-FMK) or by conditional knockout of Bim in CD19^+^ B-lineage cells, TNFα-treated pro-B cells failed to exhibit any decline in λ5 protein expression (Fig.3C). Therefore, the decline seen in λ5 levels in pro-B cells upon TNFα treatment was due to selective death of high λ5-expressing pro-B cells and increased resistance of pro-B cells with lower λ5 levels to apoptosis.

### λ5-deficient young pro-B cells are resistant to TNFα-mediated apoptosis

**Fig 4:**
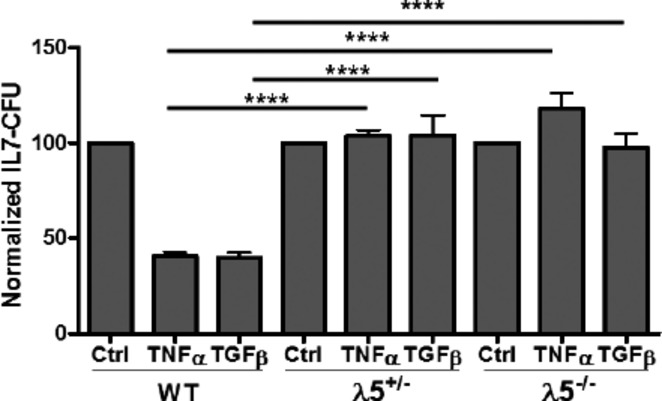
Low λ5 increases survival in pro-B cells challenged with either TNFα or TGFβ. Unfractionated bone marrow cells (1 × 10^6^ ml^−1^) from wild-type (WT), λ5 heterozygous deficient, or λ5-homozygous-deficient 2- to 3-month-old B6 mice were cultured with either 10 ng ml^−1^ rmTNFα or 10 ng ml^−1^ rmTGFβ in IL-7 CFU assays. Colonies were counted at 8 days. Normalized IL-7 CFU values were obtained in 6–12 individual experiments. Statistical significance (two-tailed Student's *t*-test): ****, *P* < 0.0001.

Pro-B cells, low in λ5 proteins in young adults, were also more resistant to cytokine-induced apoptosis. We employed young adult mice that were either homozygous (λ5^−/−^) or heterozygous (λ5^+/−^) deficient in λ5 genes, the latter having one-half of normal wild-type λ5 levels in pro-B cells (cytoplasmic staining or Western blot analysis; data not shown). As shown in Fig.4, both heterozygous and homozygous λ5-deficient bone marrow B-cell precursors were highly resistant to TNFα- and TGFβ-mediated apoptosis in IL-7 CFU assays. The TNFα receptor TNF-R1, as well as the TGFβ1 and TGFβ2 receptors, was maintained in pro-B cells from young adult λ5 KO mice and in aged mice compared to young adult wild-type controls as determined by quantitative PCR (data not shown).

### Aged B cells promote the loss of λ5^high^ pro-B cells *in vivo*

Given previous reports that depletion of B cells in old mice led to rejuvenation of B lymphopoiesis in bone marrow (Keren *et al*., 2011a,b), we addressed the capacity of B cells from old mice to reduce pro-B cells, and in particular, those with higher λ5 expression, *in vivo*. Most B-cell subsets from old mice, including FO B cells and ABC, produce the pro-inflammatory cytokine TNFα (Frasca *et al*., 2012; Ratliff *et al*., 2013). Age-associated B cells are increased in the bone marrow of aged mice even as ‘FO-like’/recirculating B cells are reduced (Hao *et al*., 2011; Ratliff *et al*., 2013).

Adoptive transfer of either ABC or FO B cells (∼10^7^) from aged (2 years old) B6 (Ly5.2) mice into young (2 months old) B6 (Ly5.1) unmanipulated recipients did not alter levels of pro-B cells approximately 1 month post-transfer (Pro-B cells in bone marrow: 1.1 ± 0.1% in PBS controls; 1.6 ± 0.3% with old ABC transfers; 1.1 ± 0.2% with old FO transfers with four recipients per group). Nor was cytoplasmic λ5 expression changed significantly as determined by fluorescence flow cytometry in pro-B cells (ABC transfers: λ5 mean fluorescence intensity (MFI) was 117 ± 11% of control values; for FO transfers λ5 MFI was 111 ± 9% of controls). Notably, levels of donor derived B cells, either ABC or FO-like, within the recipient bone marrow were only ∼0.2%.

Consequently, the bone marrow ratios of donor aged B cells to young recipient ‘FO-like’/recirculating B cells were found to be quite low (e.g., <1:40). In our prior studies *in vitro*, young adult B cells, through an IL-10-dependent mechanism, were capable of preventing the apoptosis of pro-B cells caused by aged B cells. Indeed, to overcome this effect, aged B cells (e.g., ABC) were required to be present at roughly comparable levels to mature young adult B cells if increased apoptosis in pro-B cells was to be seen (Ratliff *et al*., 2013). Therefore, the very low levels of aged B cells achieved in the bone marrow of aged B cells (Ly5.1) into Ly5.2 young adults would not be expected to affect B lymphopoiesis.

To more similarly replicate the bone marrow B-cell composition seen in aged bone marrow, where ABC often were in the majority among B cells, we utilized a previously described model where ABC or splenic FO B cells from aged mice were adoptively transferred into young adult RAG-2 KO mice (Ratliff *et al*., 2013). This model takes advantage of several unique features: first, the homeostatic expansion of B cells that occurs upon transfer into the RAG-2 KO immunodeficient environment and second, the capacity of FO B cells to generate ABC upon activation and proliferation *in vivo* (Hao *et al*., 2011).

Recovery of donor B cells, upon transfer of either old ABC or FO B cells, in RAG-2 KO spleens was somewhat higher (∼20%) compared to that seen upon B-cell transfer to unmanipulated wild-type recipients (∼5–10%, data not shown; Hao *et al*., 2011). Upon adoptive transfer of ABC into RAG-2 KO mice, at 4 weeks, 41% of splenic B cells were intermediate/high in CD21/35, but CD23^−^ consistent with an MZ (marginal zone)-like phenotype, but the majority (58%) was ABC with only 1% of FO phenotype (Table1). Transfer of old or young FO B cells into RAG-2 KO recipients led to generation of 10% ABC and 65% MZ-like (old) and 30% ABC and 89% MZ-like (young) B cells in spleen, with only 1% retaining the FO phenotype.

**Table 1:** Transfer of old age-associated B cells and follicular B cells repopulate RAG-2 KO mice

Donor cells	RAG-2 KO recipient spleen	RAG-2 KO recipient BM
ABC (old)†		
B cells per 10^6^ Transferred	16 ± 0.1	1.6 ± 0.2
Total B cells (×10^6^)	27 ± 0.1	2.7 ± 0.7
	58% ABC/41% MZ‡/1% FO	>90% ABC/<10% FO
FO (Old)†		
B cells per 10^6^ transferred	13 ± 3	0.3 ± 0.1
Total B cells (×10^6^)	122 ± 26	2.9 ± 0.1
	10% ABC/89% MZ‡/1% FO	55% ABC/45% FO
FO (Young)†		
B cells per 10^6^ transferred	19 ± 6	0.4 ± 0.1
Total B cells (×10^6^)	84 ± 27	2.0 ± 0.3
	30% ABC/65% MZ‡/5% FO	53% ABC/47% FO

† 1.7 × 10^6^ age-associated B cells (ABC) and 4.4–9.4 × 10^6^ young or old follicular (FO) were transferred i.v. into unmanipulated RAG-2 KO recipients. Splenic and bone marrow B cells were counted and characterized 1 month post-transfer.

‡ MZ B cells were CD23^−^, but include those with intermediate and high CD21/35 levels.

Adoptive transfer of ABC (or FO) B cells from old mice into RAG-2 KO mice led to levels of B cells within the bone marrow which were ∼10-fold higher than were seen when aged B cells were transferred into wild-type young mice. In the bone marrow, ABC transfer resulted in essentially only ABC B cells seen in bone marrow (>90%); old FO or young FO B cells transferred into RAG-2 KO mice led to roughly similar representation of ABC vs. FO B cells (Table1). The increased number of ABC B cells in the bone marrow of RAG-2 recipients of either ABC or splenic FO B cells mimicked the predominance of ABC B cells in bone marrow often seen in wild-type aged mice (Ratliff *et al*., 2013).

**Fig 5:**
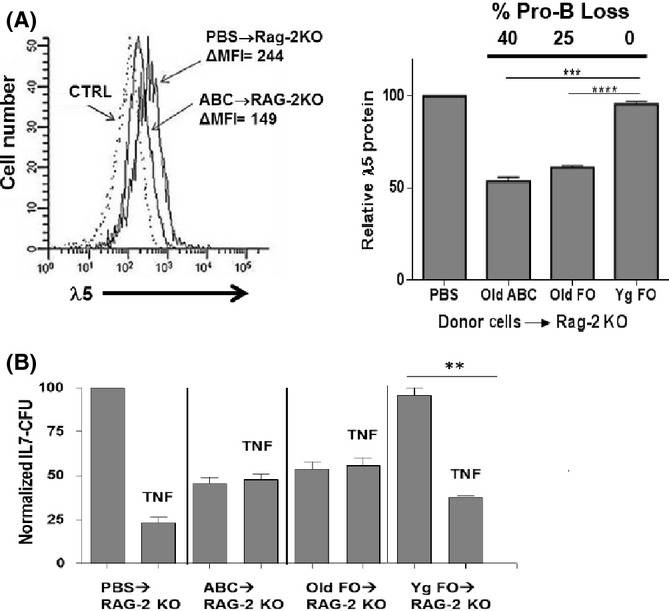
Residual pro-B cells that remain in RAG-2 KO bone marrow after adoptive transfer of old B cells are low in surrogate light chain λ5 and resist TNFα-induced apoptosis. (A, left) Representative analysis of cytoplasmic λ5 protein, detected by fluorescent staining with LM34 mAb, in pro-B cells from B6 RAG-2 KO mice either untreated (PBS injection only) or 4 weeks after adoptive transfer of 1.7 × 10^6^ splenic ABC, pooled from 3 old B6 mice (22–31 months of age). (A, right) Cumulative data is shown on pro-B-cell loss and λ5 protein expression for B6 RAG-2 KO recipients of either PBS, old B6 splenic ABC, or young or old B6 splenic follicular B cells (four mice per group) 1 month after adoptive transfer. Pro-B cell loss in the recipient bone marrow is also indicated for each group. Relative pro-B λ5 protein was calculated as the λ5 Δmean fluorescence intensity (MFI) for each group divided by the λ5 ΔMFI for the pro-B cells in PBS-treated control RAG-2 KO mice (100%). (B) Bone marrow cells from the adoptively transferred mice in (A) were cultured (1 × 10^6^ ml^−1^) in the presence/absence of optimal concentrations (10 ng ml^−1^) of rmTNFα in IL-7 CFU assays. Normalized IL-7 CFU for each experimental group were reported relative to the IL-7 CFU numbers for the control group (100%). Statistical analysis (two-tailed Student's *t*-test): **P < 0.05; ***P < 0.01; ****P < 0.001.

As we have reported (Ratliff *et al*., 2013): (i) transfer of ABC into RAG-2 KO recipients resulted in ∼40% loss of pro-B cells 1 month post-transfer; (ii) transfer of old FO B cells into RAG-2 KO recipients resulted in similar numbers of ABC and FO B cells in the bone marrow and an ∼25% loss of pro-B cells; and (iii) as control, no effects of transfer of young splenic FO B cells into RAG-2 KO recipients on pro-B cells were observed. We have now analyzed the expression of λ5 protein in the remaining pro-B cells in RAG-2 KO mice after transfer of ABC or old FO B cells. As shown in Fig.5A, λ5 protein expression in the remaining pro-B cells was diminished by approximately 40–50% in RAG-2 KO recipients of ABC and old FO B cells. No changes in λ5 protein were seen in pro-B cells from RAG-2 KO mice injected with FO B cells from young mice (Fig.5A). These results are consistent with the view that, *in vivo*, old B cells can induce the preferential loss of those pro-B cells which have higher levels of λ5 protein.

### Residual λ5^low^ pro-B cells in RAG-2 KO mice given old B cells are resistant to TNFα-induced apoptosis

The residual bone marrow pro-B cells in ABC into RAG-2 KO mice were tested to determine whether they would undergo apoptosis *in vitro*. The remaining IL-7 CFU-generating pro-B cells from ABC into RAG-2 KO mice were resistant to concentrations of TNFα which eliminated ∼80% of IL-7 CFU from PBS into RAG-2 KO bone marrow (Fig.5B). Similar resistance to TNFα-induced apoptosis was seen with pro-B cells from RAG-2 KO mice given old FO (which generated ABC, Table1). These findings indicated that the loss of pro-B cells *in vivo* mediated by aged B cells led to reduced λ5 expression and resistance to apoptosis.

### B cells from old mice, as well as young mice deficient in λ5, show increased reactivity to phosphorylcholine

**Fig 6:**
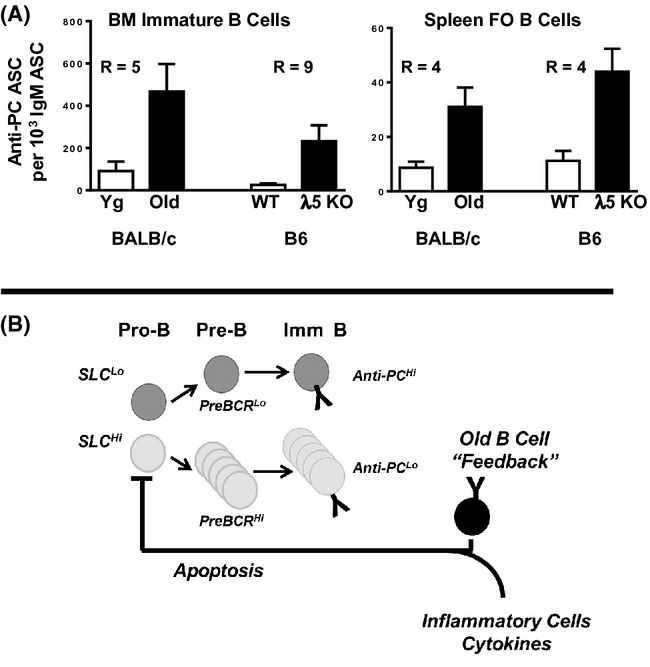
Deficiency in λ5 expression in old and young mice increases B-cell reactivity to phosphorylcholine. (A) Immature bone marrow B cells and splenic follicular (FO) B cells from young and aged BALB/c mice were cultured with lipopolysaccharide (LPS) and antibody-secreting cells (ASC) were assayed by ELISpot on phosphorylcholine (PC)-BSA and anti-μ coated plates as per Experimental Procedures. For BALB/c immature B cells, *N* = 6 young and 10 old mice; for BALB/c FO B cells, *N* = 11 young and 10 old mice. The extent of pre-B-cell loss in the old BALB/c mice used ranged from 11% to 76% (average, 59% ± 7% SEM), where four of the ten mice were severely depleted of pre-B cells (72–76%). Similarly, bone marrow B cells and splenic FO B cells were isolated from young adult λ5 KO and wild-type mice, LPS stimulated, and PC-reactive ASC determined. For bone marrow λ5 KO B cells, *N* = 11; for wild-type B cells, *N* = 10; for λ5 KO FO B cells and wild-type FO B cell groups, *N* = 3 and 4, respectively. R values shown are the ratio of the two groups indicated. P values between each pair of groups were all <0.03 using Mann–Whitney *U*-test and/or Student's *t*-test (with Welch's correction). (B) In our model, ‘pro-inflammatory’ conditions in old bone marrow, including an increase in TNFα-producing B cells, promote the apoptotic death of pro-B cells in the bone marrow. However, as pro-B cells that undergo cytokine-induced apoptosis are relatively high in surrogate light chain (SLC), this negative selection results in a reduced pro-B-cell pool with relatively low SLC expression. When B-cell precursors are ‘SLC^low^’, those new B cells which develop in bone marrow and which go on to populate the spleen show enhanced expression of autoreactive anti-PC antibodies.

Given the importance of SLC as a component of the preBCR, we asked whether low λ5 expression affected the ‘readout’ of the antibody repertoire of newly derived B cells. Previously, old mice have been shown to have increased frequencies of immature bone marrow B cells responsive to the self (and bacterial) epitope, PC (Zharhary & Klinman, 1986; Riley *et al*., 1989). As shown in Fig.6A, bone marrow immature B cells (surface IgM^+^ AA4.1^+^ CD19^+^) from aged BALB/c mice cultured with lipopolysaccharide (LPS) generated PC-reactive antibody-secreting cells (ASC) which were ∼5-fold greater compared to young immature B cells. The old BALB/c mice used in these experiments showed variable deficiency in B lymphopoiesis, with loss of pre-B cells ranging from 11% to 76% (average, 59% ± 7% SEM, *n* = 10). The majority of old mice were more ‘moderate’ in their pre-B-cell loss with only four of ten being ‘severe’ (72–76%). There was no apparent significant correlation between increased PC reactivity among immature B cells in the aged mice and extent of pre-B-cell loss within the bone marrow, albeit this may reflect the small sample sizes. In part, the extensive reactivity to PC of immature B cells and their antibodies in old mice may reflect the heightened polyreactivity and broadened V-region usage seen in anti-PC antibodies derived from old compared to young adult mice (Riley *et al*., 1989; Nicoletti *et al*., 1991).

To test the hypothesis that reduced λ5 expression was sufficient to alter the reactivity of new B cells with PC, bone marrow B cells from young adult λ5 KO mice were cultured with LPS and anti-PC ASC determined. As shown in Fig.6A, bone marrow B cells from young λ5 KO mice generated ∼ninefold more PC-reactive ASC than did bone marrow B cells from young wild-type controls.

Not only are immature B cells in the bone marrow of old mice more reactive with PC, but so too are splenic B cells (Zharhary & Klinman, 1986). Reactivity to PC of LPS-stimulated mature B cells was less than that observed for immature B cells, consistent with increased multireactivity/cross-reactivity of emergent B cells in the bone marrow (Grandien *et al*., 1994). Splenic FO B cells (IgM^+^ CD23^+^ CD21/35^+^ CD43/S7^−^ AA4.1^−^ CD19^+^) from aged mice, upon LPS stimulation, generated higher numbers of PC-reactive ASC (∼4-fold) compared to young FO B cells (Fig.6A). Splenic FO B cells from λ5 KO mice also generated ∼fourfold more ASC whose antibody was reactive with PC compared to wild-type splenic FO B cells (Fig.6A).

## Discussion

In old age, we have shown that pro-B cells are decreased and levels of SLC, both λ5 and VpreB, are diminished (Sherwood *et al*., 1998, 2000; Alter-Wolf *et al*., 2009). This may have significant ramifications for the proper functioning of the preBCR checkpoint and ‘readout’ of the antibody repertoire in newly generated B cells. Here, we have demonstrated that (i) aged mice have a residual population of pro-B cells that is relatively resistant to apoptosis; (ii) this resistance to apoptosis is dependent upon reduction in λ5 protein; (iii) aged B cells can modulate loss of pro-B cells and the bias to λ5^low^ pro-B cells *in vivo*; and (iv) reduced λ5 in B-cell precursors, either in old mice or λ5-deficient young mice, coincides with increased reactivity of B cells to the self/bacterial antigen PC.

### In old age, pro-B cells with higher SLC expression are preferentially deleted, resulting in the ‘SLC low’ phenotype of residual bone marrow pro-B cells

Both TNFα- and TGFβ-induced apoptosis in B-cell precursors was shown to be dependent on the normal expression of λ5. Indeed, it was surprising that even when λ5 protein levels were reduced by only one-half, as was seen with pro-B cells from young λ5 heterozygous mice, B-cell precursors remained as resistant to apoptosis as was seen with homozygous λ5 KO mice. The residual pro-B cells in aged mice also often show levels of λ5 protein which are reduced by half (or more), and these pro-B cells also showed relatively high resistance to apoptosis. Therefore, the apoptotic resistance of pro-B cells in aged bone marrow is likely the direct consequence of reduced SLC expression.

The signaling pathways by which SLC expression regulate apoptosis are not yet understood. On pro-B cells, SLC is bound to cadherin 17 (BILL cadherin; Ohnishi *et al*., 2000) and the cadherin17/SLC complex is associated with several, as yet uncharacterized, proteins of 200, 105, and 35–65 kDa (Karasuyama *et al*., 1993; Ohnishi *et al*., 2000). Conceivably, these proteins may be involved in signaling, and this could be altered when cadherin 17/SLC complex is reduced. While the possible mechanisms of cadherin 17/SLC complexes in regulating apoptotic sensitivity are beyond the scope of this report, in preliminary experiments, we have observed that (i) cadherin17 is normally expressed on the surfaces of old pro-B cells and young pro-B cells deficient in λ5 SLC; (ii) anticadherin 17 antibody cross-linking of cadherin 17 on partially λ5-deficient old pro-B cells and fully λ5-deficient λ5 KO pro-B cells restores their susceptibility to TNFα-induced apoptosis; and (iii) the cadherin 17/SLC complex affects relative levels of functional pro-apoptotic Bim protein in pro-B cells (Ratliff, M., Chirinos, A., Khan, W.N., Blomberg, B.B., and Riley, R.L., manuscript in preparation). Therefore, it is possible that the relative levels of cadherin 17/SLC have a direct effect on sensitivity to apoptosis in pro-B cells.

### Old B cells can promote loss of λ5^high^ pro-B cells in the bone marrow *in vivo*

We were unable to transfer enough aged B cells into unmanipulated wild-type recipients so that old-derived B cells (e.g., ABC) would represent a fraction of total B cells sufficient to downregulate B lymphopoiesis. However, when adoptive transfer of far fewer (two- to threefold less) ABC or FO B cells from aged mice into RAG-2 KO mice was performed, pro-B cells were depleted within the bone marrow (Ratliff *et al*., 2013). Like pro-B cells in aged mice, the remaining pro-B cells in RAG-2 KO mice after old ABC or FO transfer demonstrated lower expression of λ5 protein. Most importantly, the remaining pro-B cells in these transplanted mice were now resistant to TNFα-induced apoptosis, consistent with their low λ5 protein phenotype. This provides a ‘proof-of-principle’ that old B cells can have a negative effect on pro-B-cell numbers within the bone marrow and also can alter both apoptotic susceptibility and λ5 protein expression in pro-B cells. However, as these old B cells are placed in the lymphopenic RAG-2 KO microenvironment, this may alter their properties and conclusions from these experiments must be interpreted with caution.

Previous reports have demonstrated that depletion of B cells *in vivo* in old mice results in revitalization of B lymphopoiesis within the bone marrow (Keren *et al*., 2011a,b). These studies suggest a ‘feedback’ homeostatic mechanism where B cells in old mice play important roles in limiting further B-cell development within the bone marrow. Our results suggest that old B cells, in particular ABC, may impair pro-B-cell survival. However, apoptosis may act preferentially to eliminate pro-B cells with relatively high SLC, leaving a reduced pool of pro-B cells with lower SLC content in old mice. Further studies directed at elucidating procedures to specifically delete ABC (and other B-cell subsets) from normal aged mice are required to determine the relative effects of ABC as well as other B cells on B lymphopoiesis in old age.

While B cells in old mice do produce TNFα, we recognize that there are likely additional sources of TNFα, as well as other cytokines and biomediators, which may increase the ‘apoptotic stress’ within the bone marrow in old age. For example, we have previously shown that TNFα-producing NK cells are elevated in the bone marrow of aged mice (King *et al*., 2009). In old age, a variety of cells, including adipose cells, macrophages, NK cells, as well as B cells produce TNFα (Wu *et al*., 2007; King *et al*., 2009; Frasca *et al*., 2012; Ratliff *et al*., 2013). Dissecting the relative importance of each cell source and cytokine are studies which merit further efforts. However, as shown herein, TNFα-producing B cells, including ABC, have the potential to cause reduced pro-B cells characterized by decreased λ5 expression and resistance to apoptosis.

### Low surrogate light chain results in increased anti-phosphorylcholine reactivity in both immature and mature B cells in old age

It has been known that the frequency of B cells reactive with the PC epitope is increased significantly among newly formed bone marrow B cells as well as in splenic B cells in old age (Zharhary & Klinman, 1986; Riley *et al*., 1989). We made use of this finding to assess whether low λ5 expression coincided with increased PC reactivity in B cells. We demonstrated that immature B cells in the bone marrow of aged mice (where λ5 SLC is reduced) were indeed increased in PC reactivity. Furthermore, mature splenic FO B cells from aged mice also showed similarly increased PC reactivity. Importantly, bone marrow B cells as well as splenic FO B cells from young adult λ5 KO mice also were enriched for anti-PC reactivity. This suggests that reduced SLC levels are sufficient to bias B-cell repertoires toward PC reactivity. Keenan *et al*. (2008) have shown heightened autoreactivity, for example, to DNA and nuclear antigens, in B cells that develop in SLC-deficient mice. Our data are consistent with this view and now suggest that B cells reactive to PC, an apoptotic self-antigen, continue to be selected for B-cell maturation when SLC is reduced.

We suggest that, as illustrated in Fig.6B, in old mice ‘pro-inflammatory’ TNFα-producing B cells, including ABC, within the bone marrow increase apoptosis and this leads to the preferential death of SLC^high^ pro-B cells within the bone marrow. This increased ‘apoptotic stress’ likely also is exacerbated by other inflammatory cells and cytokines as discussed above. Consequently, further B-cell development not only is diminished, but occurs under predominantly ‘SLC low’ conditions. An ‘SLC low’ pathway of B-cell development, by altering preBCR-mediated V_H_ selection, may affect the readout of the antibody repertoire, as shown by several studies (Ye *et al*., 1996; ten Boekel *et al*., 1997; Kline *et al*., 1998). Whether pre-B cells with V_H_ genes which encode the heavy chains of anti-PC antibodies are less dependent upon SLC levels and preBCR selection is not known. Nevertheless, as shown herein, low SLC expression impacts not only the newly generated, but the naïve peripheral B-cell repertoire. This has implications for immune competence with regard to recognition of foreign antigens for immune defense and vaccine responses as well as for the increased autoreactivity that is seen in old age.

## Experimental procedures

### Mice

Young (2–4 months old) and aged (24–31 months old) C57BL/6 and young (2–4 months old) and aged (22–24 months old) BALB/c female mice were obtained from the National Institute on Aging. B6 (Cg)-Rag2^tm1.1Cgn^/J (B6 RAG-2 knockout) mice, B6.129S2-Igll1^tm1Cgn^/J (λ5 gene knockout) mice were originally obtained from the Jackson Laboratories (Bar Harbor, ME, USA) and B6.SJL-Ptpc^a^ Pepc^b^/BoyJ (B6 Ly5.1) mice (2 months old) were also obtained from the Jackson Laboratories. B-lineage-specific Bim-deficient mice (B-Bim^−/−^) were generated by crossing Bim^fl/fl^ (Takeuchi *et al*., 2005) with CD19Cre^+/−^ mice (Rickert *et al*., 1997). All the B-Bim^−/−^ mice analyzed were heterozygous for CD19 with no effect on B-cell developmental phenotype (Rickert *et al*., 1997). Animal protocols were approved by the University of Miami Institutional Animal Care and Use Committee (IACUC).

### Fluorescent cell staining and analysis

Bone marrow cells were stained for IgM, CD43/S7, CD19, CD2, as described in Ratliff *et al*., 2013. Anti-CD179b-biotin (clone LM34, anti-λ5), followed by streptavidin-PerCP, all from BD Biosciences (San Jose, CA, USA) was used to stain for λ5 protein. For the cytoplasmic staining of μ chain and λ5, cells were stained for surface markers, permeabilized using BD Cytofix/Cytoperm (BD Biosciences), washed with PermWash (BD Biosciences), and followed by cytoplasmic stain addition. In some experiments, cells were also stained for either Ly5.1 or Ly5.2 (clones A20 and 104; BD Biosciences) or with anti-CD138 (clone 281-2; BD Biosciences) appropriately fluorescently labeled. Cells were analyzed on either an LSR-Fortessa, or LSR II or a FACS Canto fluorescence flow cytometer (BD Biosciences).

### Identification and isolation of B-cell precursors

Pre-B cells (surface CD2^+^ IgM^−^ CD19^+)^ and pro-B cells (surface CD2^−^ IgM^−^ CD19^+^) were identified in bone marrow or in IL-7 CFU by fluorescence flow cytometry. Differential expression of CD2 on pro-B vs. pre-B cells has been previously reported (Sen *et al*., 1990). The CD2^−^ IgM^−^ CD19^+^ pro-B cells were uniformly CD43/S7^+^ AA4.1^+^, <10% cytoplasmic μ^+^, but >80% cytoplasmic λ5^+^ and surface c-kit^+^ as would be expected for pro-B Hardy Fractions B, C, and pre-B I (pro-B) cells. CD2^+^ IgM^−^ CD19^+^ pre-B cells were uniformly negative for CD43/S7, but positive for AA4.1; uniformly cytoplasmic μ^+^, <10% cytoplasmic λ5^+^ and <5% c-kit^+^ as expected.

Pro-B cells express readily detectable λ5 protein in their cytoplasm (Sherwood *et al*., 1998). Relative levels (ΔMFI) of cytoplasmic λ5 protein expression were determined based on the MFI minus background/control staining MFI for a given sample. This was generally expressed as a fraction of that seen in control (e.g., young adult) pro-B cells whose MFI was set to ‘1.0’.

In some experiments, total CD19^+^ B-lineage cells from bone marrow were isolated by magnetic bead (MACS) sorting using anti-CD19-PE or anti-CD19-APC (BD Biosciences) and anti-PE or anti-APC microbeads (Miltenyi Biotec, Auburn, CA, USA) following the miniMACS protocols. CD19^+^ cells were purified over magnetic columns to a purity of >97%.

### Cell culture

Femur and tibia pairs were flushed to harvest cells from the bone marrow as previously described (Riley *et al*., 1991). Single cell suspensions were washed and counted for use in flow cytometry, cell sorting, or cell culture. When obtaining IL-7 expanded B-cell precursors, bone marrow cells were cultured at 1–2 × 10^6^ cells ml^−1^ with 5 ng ml^−1^ rmIL-7 in RPMI-1640 (Invitrogen, Carlsbad, CA, USA) supplemented with 10% FCS (low endotoxin; Sigma–Aldrich, St. Louis, MO, USA) plus 1% penicillin-streptomycin, 1% glutamine, and 5 × 10^−5^ m 2-mercaptoethanol.

In analysis of apoptotic mechanisms, a caspase 3 inhibitor, Z-DEVD-FMK (BD Biosciences), was used to block caspase 3 activity with Z-FA-FMK serving as negative control (BD Biosciences) (Ratliff *et al*., 2013). Cells were preincubated with inhibitor or control for 30 min. Cells were cultured for the indicated times and assessed for intracellular λ5 protein as described above. Added inhibitor or control was included to maintain original concentrations (50 mm) during culture.

### IL-7 CFU assay

B-cell precursors, either in unfractionated (whole) bone marrow (WBM) or as CD19^+^ B-lineage cells isolated by magnetic sorting were cultured in semi-solid methylcellulose media to derive pro-B/pre-B-cell colonies as previously described (Riley *et al*., 1991; Ratliff *et al*., 2013). In each experiment, IL-7 CFU assays were performed in triplicate for each group. Typically, IL-7 CFU in control cultures ranged from ∼500 to as much as 3000, depending upon the experiment. As a consequence of IL-7 CFU control culture variability in colony counts for individual experiments, IL-7 CFU were normalized to that of the control cultures (set at 100%) in each experiment. The averages of these normalized IL-7 CFU data were then used to compare results from multiple individual experiments.

### Isolation of age-associated B cells and follicular B cells and their adoptive transfer to wild-type or RAG-2 KO recipients

Age-associated B cells and FO B cells were isolated as described in Ratliff *et al*. (2013) (97–99% purity). Age-associated B cells or FO B cells, from aged or young B6 mice, were adoptively transferred (i.v.) into unmanipulated young adult RAG-2 KO or wild-type Ly5.1 B6 mice at cell doses ranging from 1.7 to 10 × 10^6^. After 4 weeks, bone marrow was analyzed to assess pro-B-cell levels, cytoplasmic λ5, and responses to TNFα *in vitro*.

### ELISpot assay

Immature bone marrow B cells (IgM^+^ AA4.1^+^ CD19^+^) were isolated by fluorescence cell sorting from individual young and old BALB/c mice. Old mice may have elevated B1 B cells and plasma cells/blasts, the latter of which may express AA4.1 (Chevrier *et al*., 2009). Note that CD138 (Syndecan-1) is normally expressed by many immature B cells (Sanderson *et al*., 1989) as well as plasma blasts/plasma cells; therefore, depletion protocols dependent upon CD138 were not practical. We confirmed that, as expected (Gulley *et al*., 1988; Soro *et al*., 1999), ∼75–95% of CD138^high^ plasma cells/plasma blasts coexpressed CD43/S7 (data not shown). Therefore, in 7 of 10 old immature B-cell sorts, CD43/S7^+^ B cells were also gated out, thereby avoiding both B1 B cells and most plasma cells/plasma blasts. No differences were seen in the results obtained either with or without CD43/S7^+^ B-cell depletion.

For ELISpot analysis, approximately 1–2 × 10^6^ B cells were cultured in RPMI 1640 supplemented with 10% FCS/1% glutamine/2 × 10^−5^ 2-mercaptoethanol/1% penicillin–streptomycin. Cells were challenged with 5–10 μg ml^−1^ LPS (*E.coli* O55:B5; Sigma–Aldrich). After 5 days, cells were harvested and analyzed by ELISpot. Recovered cells, from ∼5 × 10^2^ to 2 × 10^5^ for anti-PC assays and ∼1 × 10^2^ to 1 × 10^4^ for total IgM assays, were transferred to 96-well microtiter plates precoated with either phosphorylcholine (PC_2_)-bovine serum albumin (BSA) (Biosearch Technologies, Petaluma, CA, USA) or anti-μ polyclonal antibody (goat; Jackson ImmunoResearch, West Grove, PA, USA).

Plates were developed 24 h later with HRP-goat anti-mouse κ light chain antibody (Southern Biotech, Birmingham, AL) and 3-amino-9-ethylcarbazole (AEC) substrate (BD Biosciences). Plates were read in an ImmunoSpot reader (Cellular Technology Ltd., Cleveland, OH, USA). The frequency of ASC was calculated based on the linear regression line for ASC vs. cell dilution. Lipopolysaccharide stimulation showed experimental variation with IgM ASC trending ∼50% lower for aged immature B cells compared to young controls. Therefore, in each experiment, anti-PC ASC were normalized to total IgM ASC to correct for differences in LPS stimulation. In the absence of LPS, IgM responses were only ∼7–14% of that seen upon LPS stimulation.

Old and young FO splenic B cells were isolated by fluorescence cell sorting as IgM^+^ AA4.1^−^ CD43/S7^−^ CD23^+^ CD21/35^+^ CD19^+^ cells. Follicular B cells were also isolated from spleens of B6 and λ5 KO mice as CD23^+^ cells by magnetic bead sorting using biotin rat anti-mouse CD23 antibody (BD Biosciences) with antistreptavidin microbeads and MS or LS Columns (Miltenyi Biotec) or by fluorescent cell sorting. Bone marrow B cells (IgM^+^) were used from these mice as a source of immature B cells with sorting via APC rat anti-mouse IgM (BD Biosciences) with anti-APC microbeads and MS or LS Columns (Miltenyi Biotec).

### Statistical analysis

Groups were compared by two-tailed Student's *t*-test or Mann–Whitney *U*-test with p values shown.
